# Breast Implants and Breast Cancer

**Published:** 2012-07

**Authors:** Davood Mehrabani, Ail Manafi

**Affiliations:** 1 Stem call and Transgenic Technology Research Center, Department of Pathology, Shiraz university of Medical sciences, Shiraz, Iran; 2 Department of Plastic Surgery, Tehran University of Medical Sciences, Tehran, Iran

Brest cancer is still the most common cancer among Female in the world and is prevalent in different parts of Iran too.^1^ Silicone implants were applied for augmentation of the breast for aesthetic goals or post-lumpectomy or mastectomy because of malignancies in the tissue.^2^ The implant was shown to have inflammatory, fibro Proliferate response and connective tissue disorders.^3^^,^^4^

In January issue of the journal in 2012, three articles were published regarding breast cancer and breast implantation, which are discussed in this paper. Mehrabani *et al.*^5^ describe incidence of breast cancer in southern Iran with valuable information considering the risk factors. But it lacks data after year 2006 while the face of this cancer may change by time. Therefore, it is recommended that new information on breast cancer from 2007 to 2012 are published in future issue with a more detailed discussion of data in the same region as well as other cities of the country.

In another study of this issue by Ravi-Kumar *et al.*^6^ on anaplastic large cell lymphoma (ALCL) associated with breast implants, they described a 42 years old woman with bilateral breast augmentation for aesthetic purposes who had poor healing at the surgical site. Excisional biopsy revealed ALCL in the tissue. Even it is a really good case report in their region but this report lacks the treatment outcome of the patient and did not explain why the patient was not transferred to an academic metropolitan university hospital. It was more attractive for readers if they presented any data on the frequency of breast implants due to tissue malignancies in the country. Surgical out-come was not so clear too. It should also be mentioned that the authors did not reach a reasonable conclusion and comments for the readers.

In the third paper of that issue, Rajabiani *et al.*^7^ demonstrated a case of ALCL associated with breast implant. This article provided good information on breast implants in malignancies for plastic surgeons while the report was new in their locality like the previous report. They both efficiently discussed the presentations of the case and the therapeutic measures. This report lacks discussion on clinicopathological features of implant associated ALCL and have not denoted to the treatment outcome of the patient even the conclusion and comments cover the expectations of the readers.

Therefore, all breast tissues which are removed for implant related complications should carefully be examined by a pathologist and they must be aware that this entity may easily be misdiagnosed on histopathological examinations.

## CONFLICT OF INTEREST

The authors declare no conflict of interest.
